# Serial pulmonary function tests to diagnose COPD in chronic heart failure

**DOI:** 10.1186/s40247-014-0012-5

**Published:** 2014-09-25

**Authors:** Armine G Minasian, Frank JJ van den Elshout, PN Richard Dekhuijzen, Petra JE Vos, Frank F Willems, Paul JPC van den Bergh, Yvonne F Heijdra

**Affiliations:** Department of Pulmonary Diseases, Rijnstate Hospital, Wagnerlaan 55, Arnhem, 6815, AD The Netherlands; Department of Pulmonary Diseases, Radboud University Nijmegen Medical Centre, Geert Grooteplein-Zuid 10, Nijmegen, 6525, GA The Netherlands; Department of Cardiology, Rijnstate Hospital, Wagnerlaan 55, Arnhem, 6815, AD The Netherlands; Department of Pulmonary Diseases, Rijnstate Hospital, Arnhem, 6800, TA The Netherlands

**Keywords:** Chronic obstructive pulmonary disease, Chronic heart failure, Prevalence, Serial pulmonary function tests, Underdiagnosis, Overdiagnosis

## Abstract

**Background:**

It is unknown whether serial pulmonary function tests are necessary for the correct diagnosis of chronic obstructive pulmonary disease (COPD) in patients with stable non-congested chronic heart failure (CHF). The aim of this study was to determine the prevalence of COPD in outpatients with stable CHF without pulmonary congestion using initial as well as confirmatory spirometry three months after treatment for COPD.

**Methods:**

Spirometry was performed in 187 outpatients with stable CHF without pulmonary congestion on chest radiograph who had a left ventricular ejection fraction < 40% (mean age 69 ± 10 years, 78% men). COPD was defined according to the Global Initiative for Chronic Obstructive Lung Disease guidelines. The diagnosis of COPD was confirmed three months after treatment with tiotropium in newly diagnosed COPD patients.

**Results:**

Using a three month follow-up spirometry to confirm initial diagnosis of de novo COPD did not change COPD prevalence significantly: 32.6% initially versus 32.1% after three months of follow-up. Only 1 of 25 (4%) patients with newly diagnosed COPD was not reproducibly obstructed at follow-up. COPD was greatly under- (19%) and overdiagnosed (32%).

**Conclusions:**

Spirometry should be used under stable and euvolemic conditions to decrease the burden of undiagnosed or overdiagnosed COPD in patients with CHF. Under these conditions, a confirmatory spirometry is unnecessary, as it does not change a newly established diagnosis of COPD in the vast majority of patients with CHF.

**Trial registration:**

ClinicalTrials.gov Identifier NCT01429376.

**Electronic supplementary material:**

The online version of this article (doi:10.1186/s40247-014-0012-5) contains supplementary material, which is available to authorized users.

## Background

Heart failure (HF) is a common clinical condition with high mortality and morbidity rates [1]. Chronic obstructive pulmonary disease (COPD) frequently coexists with HF, leading to poor prognosis as well as diagnostic and therapeutic challenges [2]-[17]. Estimates of COPD prevalence in patients with HF vary substantially between 9% and 52% in earlier reports that relied on clinical data, disease codes, or self-reported COPD for establishing the diagnosis [2]. Spirometry is considered to be the gold standard for the diagnosis of COPD [18], but is unfortunately still underutilised [19]. Studies that used spirometry have also reported varying prevalence rates of COPD (9 - 44%) depending on study design, population, and diagnostic criteria [14]-[17],[20]-[23]. Airway obstruction is a dynamic phenomenon in HF, as it may be present in congestive HF and may disappear with treatment of HF [14]. Therefore, a careful timing and interpretation of pulmonary function tests (PFTs) is required to avoid misdiagnosis and inappropriate treatment [2]. Ideally, serial PFTs should be used under stable conditions when clinically euvolemic to establish a valid diagnosis of COPD by confirming persistent airway obstruction. However, data on the need of serial pulmonary function measurements are scarce and even lacking in patients with stable chronic HF (CHF). It is therefore unknown whether a confirmatory spirometry is necessary for the correct diagnosis of COPD in patients with stable non-congested CHF.

The present study determined the prevalence of COPD in outpatients with stable CHF without pulmonary congestion using initial as well as confirmatory spirometry three months after treatment with tiotropium in patients with newly diagnosed COPD.

## Methods

### Study design and participants

All patients attending two outpatient cardiology departments of a large general hospital in The Netherlands were screened for inclusion in this prospective study between October 2009 and December 2010. In addition, existing patient lists were used to ensure that the majority of HF population had been examined for eligibility. Inclusion criteria were CHF [1] with left ventricular ejection fraction (LVEF) < 40%, New York Heart Association (NYHA) class I-IV, and age of ± 18 years. CHF was defined according to European Society of Cardiology guidelines [1]. Echocardiography was performed in patients without a recent (≤6 months) echocardiography to confirm persisting left ventricular systolic dysfunction (LVSD). Patients were classified as having stable HF in the absence of hospitalization due to progression of HF within 3?months, change in diuretics within 1?month, 3% or more weight gain within 3?days, and more than 50% increase of N-terminal pro-B natriuretic peptide (NT-pro-BNP) within 1?month when the baseline NT-pro-BNP was 100 pmol/L or higher or more than 100 pmol/L increase of NT-pro-BNP within 1?month when baseline NT-pro-BNP was below 100 pmol/L [24]. Pulmonary congestion was evaluated on standard posterior-anterior and lateral chest radiographs for the presence or absence of alveolar oedema, pleural effusion, Kerley-B lines, and/or the redistribution of pulmonary blood flow by independent radiologists who qualitatively assessed the chest radiographs with an overall clinical impression. We excluded patients who were not able to cooperate or undergo spirometry or who had asthma according to their medical chart. Other exclusion criteria were malignancy with a poor prognosis (survival < 6?months) and participation in another cardiology study. Patients who had been hospitalized in the pulmonary department in the past six weeks were included six weeks after discharge to ensure that their pulmonary function was stable at the time of spirometry testing.

### Measurements and data collection

At baseline, a first blood sample was taken for the measurement of NT-pro-BNP according to the standard methods used at the hospital laboratory. One month later, the participants visited the hospital for an interview with the investigator and several examinations, including height and weight measurement, spirometry, and a chest radiograph. In addition, a second blood sample (NT-pro-BNP) was taken to determine the stability of HF. The Minnesota Living with Heart Failure Questionnaire [25], modified Medical Research Council dyspnoea scale [26], and 10-point Borg score [27] were used to evaluate quality of life, effect of breathlessness on daily activities, and dyspnoea at rest, respectively. Additional data were collected from medical records and personal interviews. Arterial blood gas analysis was carried out in patients with severe airway obstruction to determine whether they had chronic respiratory failure [18].

Patients with newly diagnosed COPD were followed up three months after standard treatment for COPD with once-daily 18 μg tiotropium. A third blood sample (NT-pro-BNP) was taken and spirometry was repeated to confirm persistent airway obstruction characteristic of COPD and exclude asthma as much as possible. Thus only patients with persistent airway obstruction on three months of follow-up were classified as having COPD.

### Spirometry testing

Spirometry (MasterLab Pro; Jaeger; Würzburg, Germany) was performed by trained and certified operators using standard techniques and according to European Respiratory Society standards for acceptability and reproducibility [28]. The reference values of the European Community for Coal and Steel were used [28]. Subjects with airway obstruction underwent post-bronchodilator spirometry 30 minutes after inhalation of four doses of 100 μg aerosolised salbutamol and four doses of 20 μg aerosolised ipratropium via Volumatic spacer. Participants were instructed not to take bronchodilators 6?24 hours before the tests, depending on the type of bronchodilator used. At follow-up, salbutamol and ipratropium were used, as previously described, when patients discontinued the use of tiotropium > 24 hours prior to spirometry. Care was taken to match the timing of the second spirometry testing to the first to reduce variations that may occur over a 24-hour period.

### Definitions

*COPD* was defined according to the Global Initiative for Chronic Obstructive Lung Disease (GOLD) guidelines as post-bronchodilator ratio of forced expiratory volume in one second to forced vital capacity (FEV_1_/FVC) < 0.7 [18]. *COPD severity staging* was determined on the basis of FEV_1_ percent predicted according to GOLD criteria: FEV_1_ ± 80% predicted (stage I, mild), 50% ± FEV_1_ < 80% predicted (stage II, moderate), 30% ± FEV_1_ < 50% predicted (stage III, severe), and FEV_1_ < 30% predicted or FEV_1_ < 50% predicted plus chronic respiratory failure (stage IV, very severe) [18].

*Smoking status* was defined as never (<100 cigarettes in a lifetime), former (≥3 months ago), or current smoker (<3 months). *Smoking pack-years (PY)* were based only on the tobacco cigarette history and one PY was defined as smoking 20 cigarettes a day for 1 year.

*Dyspnoea* was defined as resting dyspnoea or dyspnoea at any level of exertion, *chronic cough* as cough ± 3 months prior to the study, *chronic sputum production* as regular production of sputum for ± 3 months in 2 consecutive years, and *aspecific bronchial hyperreactivity (ABHR)* as respiratory symptoms in response to perfumes, scent of baking or paint, fog, cold air, or temperature changes.

### Ethical considerations

The study was approved by the regional Research Ethics Committee Arnhem-Nijmegen in The Netherlands (2009/101, NL27798.091.09, ClinicalTrials.gov Identifier NCT01429376) and complies with the Declaration of Helsinki. All participants gave written informed consent.

### Statistical analysis

Descriptive data are presented as mean ± standard deviation (SD) or as number (%). Baseline characteristics of patients with and without COPD were compared using the independent t-test or Mann-Whitney U test for continuous variables and the chi-square or Fisher's exact test for categorical variables, as appropriate. Correlations between COPD and LVEF and between COPD and NT-pro-BNP were examined using the Pearson's and Spearman's correlation coefficient tests, respectively. Association between COPD and NYHA class was examined using the chi-square test. Statistical analyses were performed using the Statistical Package for Social Science (SPSS, version 15.0). All statistical tests were two-sided and a p-value < 0.05 was considered significant.

## Results

### Patient characteristics

**Figure 1:**
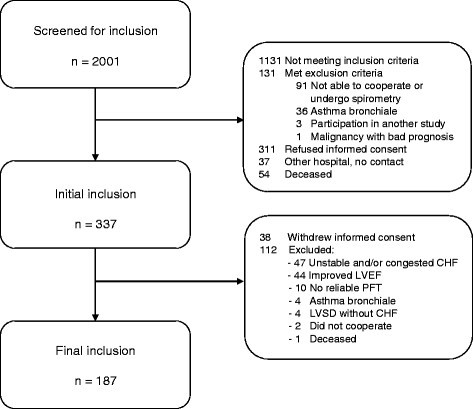
**Flow-diagram of screening and final inclusion of study participants.** CHF, chronic heart failure; LVEF, left ventricular ejection fraction; LVSD, left ventricular systolic dysfunction; PFTs, pulmonary function tests.

After screening of the entire HF population, a cohort of 337 patients with CHF was initially enrolled in this study. Thirty-eight patients withdrew informed consent and 65 patients were excluded due to several other reasons as specified in Figure 1. The remaining 234 patients were finally included of whom 187 had stable CHF without signs of pulmonary congestion. The characteristics of these patients are shown in Tables 1 and 2. Mean age and LVEF were 69 ± 10 years and 29 ± 7%, respectively, and 78% were men. The majority of patients (72%) had NYHA class II, while only 16% and 12% had NYHA class I and III/IV, respectively. Almost 60% had an ischaemic aetiology of HF. Other causes of HF were idiopathic (24%), hypertension (6%), valve disease (6%), tachycardiomyopathy (3%), and other (2%). Most patients were former or current smokers (83%) and reported symptoms of dyspnoea (82%). Other respiratory symptoms were less common (cough 36%, sputum 23%, ABHR 29%). The patients received optimised, individually tailored drug treatment as maintenance therapy for their CHF.

**Table 1:** **Characteristics of patients with and without COPD based on spirometry**

	All (n = 187)	No COPD (n = 127)	COPD (n = 60)	P-value
Age, years	69 ± 10	68 ± 11	70 ± 9	0.173
Male sex, n (%)	146 (78)	94 (74)	52 (87)	0.051
BMI, kg/m^2^	28 ± 5	29 ± 5	28 ± 5	0.064
LVEF, %	29 ± 7	29 ± 7	29 ± 7	0.401
NYHA class, %				
NYHA I-II	164 (88)	114 (90)	50 (83)	0.211
NYHA III-IV	23 (12)	13 (10)	10 (17)	0.211
Ischaemic aetiology	110 (59)	71 (56)	39 (65)	0.238
Smoking history, n (%)				0.008
Non-smoker	32 (17)	29 (23)	3 (5)	
Current smoker	23 (12)	13 (10)	10 (17)	
Former smoker	132 (71)	85 (67)	47 (78)	
PY, years	24 ± 24	21 ± 21	30 ± 28	0.016
Co-morbidity, n (%)				
Myocardial infarction	109 (58)	71 (56)	38 (63)	0.336
Atrial fibrillation	54 (29)	38 (30)	16 (27)	0.647
Hypertension	80 (43)	51 (40)	29 (48)	0.291
Diabetes mellitus	46 (25)	33 (26)	13 (22)	0.522
PCI/CABG	76 (41)	48 (38)	28 (47)	0.249
CRT/ICD	64 (34)	49 (39)	15 (25)	0.068
Medication, n (%)				
ACE-I/ARB	174 (93)	119 (94)	55 (92)	0.759
β-blockers	172 (92)	116 (91)	56 (93)	0.778
± Selective	99 (58)	66 (57)	33 (59)	0.801
± Non-selective	73 (42)	50 (43)	23 (41)	0.801
Diuretics	159 (85)	107 (84)	52 (87)	0.666
Aldosterone-antagonists	65 (35)	47 (37)	18 (30)	0.348
ICS/OCS	26 (14)	9 (7)	17 (28)	0.000
β-agonists	29 (16)	7 (6)	22 (37)	0.000
Anticholinergics	29 (16)	6 (5)	23 (38)	0.000

Data are presented as mean ± SD and number (%).

*Abbreviations:* *ACE-I/ARB* angiotensin-converting enzyme inhibitor/angiotensin receptor blocker, *BMI* body mass index, *COPD* chronic obstructive pulmonary disease, *CRT/ICD* cardiac resynchronisation therapy/implantable cardioverter defibrillator, *ICS/OCS* inhalation/oral corticosteroids, *LVEF* left ventricular ejection fraction, *NYHA* New York Heart Association, *PCI/CABG* percutaneous coronary intervention/coronary artery bypass grafting, *PY* pack-years.

**Table 2:** **Pulmonary symptoms and results of questionnaires, laboratory tests, and spirometry of patients with and without COPD based on spirometry**

	All (n = 187)	No COPD (n = 127)	COPD (n = 60)	P-value
Symptoms, n (%)				
Cough	67 (36)	38 (30)	29 (48)	0.014
Sputum	43 (23)	26 (20)	17 (28)	0.233
Dyspnoea	153 (82)	101 (80)	52 (87)	0.237
ABHR	55 (29)	29 (23)	26 (43)	0.004
Questionnaires				
MLHFQ	20 ± 17	19 ± 17	21 ± 18	0.568
MRC	1.5 ± 1.3	1.4 ± 1.3	1.7 ± 1.4	0.149
Borg	0.9 ± 1.2	0.8 ± 1.2	1.1 ± 1.3	0.036
Laboratory data				
NT-pro-BNP1, pmol/L	201 ± 289	184 ± 273	236 ± 321	0.290
NT-pro-BNP2, pmol/L	198 ± 308	179 ± 289	239 ± 345	0.250
Spirometry				
FEV_1_, L	2.5 ± 0.8	2.7 ± 0.8	2.1 ± 0.7	0.000
FEV_1_, % predicted	88 ± 21	96 ± 15	72 ± 22	0.000
FVC, L	3.7 ± 1.0	3.7 ± 1.0	3.8 ± 1.0	0.699
FVC, % predicted	102 ± 19	102 ± 17	101 ± 23	0.602
FEV_1_/FVC, %	68 ± 11	74 ± 5	55 ± 10	0.000

Data are presented as mean ± SD. Only pre-bronchodilator lung function test results are presented to make comparison between groups possible. Laboratory data 1 and 2 refer to first (at baseline) and second (one month later) blood samples, respectively.

*Abbreviations:* *ABHR* aspecific bronchial hyperreactivity, *COPD* chronic obstructive pulmonary disease, *FEV*_*1*_*/FVC* ratio of forced expiratory volume in one second to forced vital capacity, *MLHFQ* Minnesota Living with Heart Failure Questionnaire, *MRC* modified Medical Research Council dyspnoea scale, *NT-pro-BNP* N-terminal pro-B natriuretic peptide.

### COPD prevalence

Initially, 61 (32.6%) CHF patients were diagnosed with COPD based on spirometry of whom 34 had a history of obstructive lung disease (OLD). Subsequently, 27 patients with newly diagnosed COPD were followed up three months after standard treatment for COPD. Two patients were lost to follow-up; one deceased and the other withdrew informed consent. One of the remaining 25 patients no longer had airway obstruction at follow-up, which was classified as mild upon initial assessment. Thus, COPD prevalence was 32.1% [25.4-38.8%] after three months of follow-up.

**Figure 2:**
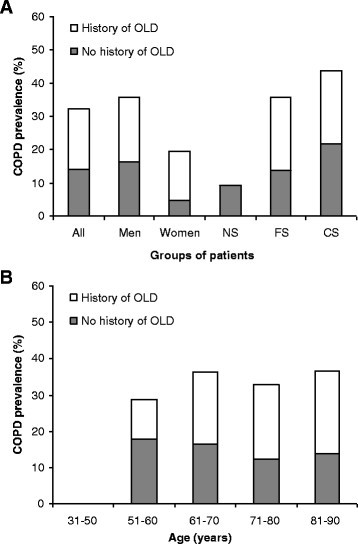
**COPD prevalence and underdiagnosis according to (A) gender and smoking status, and (B) age categories.** COPD, chronic obstructive pulmonary disease; CS, current smokers; FS, former smokers; NS, non-smokers; OLD, obstructive lung disease. The grey parts of the bar represent the proportion of patients with COPD based on spirometry who did not have a history of OLD (i.e. previously underdiagnosed patients).

COPD prevalence tended to be higher in men than women (p = 0.051). It was also higher in former (36%) and current smokers (43%) than in non-smokers (9%), with no significant differences between current and former smokers (Figure 2A). None of the 9 patients aged between 31 and 50 years were diagnosed with COPD. COPD prevalence according to other age categories is shown in Figure 2B. Most patients had mild (46.7%) or moderate (40.0%) COPD, while only 8.3% and 5.0% had severe or very severe COPD, respectively.

### Underdiagnosis and overdiagnosis of COPD

COPD was both over- and underdiagnosed (Figure 2 and Table 3). In terms of overdiagnosis, 32% (16/50) of patients with a history of OLD failed to demonstrate airway obstruction. In terms of underdiagnosis, 19% (26/137) of patients without a history of OLD were newly diagnosed with COPD based on spirometry. A history of OLD was defined as COPD (n = 43) or not further specified airway obstruction (n = 7) based on patient charts and/or general practitioner diagnosis.

**Table 3:** **Underdiagnosis and overdiagnosis of COPD**

	COPD (+)	COPD (-)	Total
History of OLD (+)	34 (18.2)	16 (8.6)	50 (26.7)
History of OLD (-)	26 (13.9)	111 (59.4)	137 (73.3)
Total	60 (32.1)	127 (67.9)	187 (100)

Data are presented as number (%).

*Abbreviations:* *COPD* chronic obstructive pulmonary disease, *OLD* obstructive lung disease.

### Determinants of COPD

Table 1 shows characteristics of patients with and without COPD based on spirometry. Pulmonary symptoms and the results of questionnaires, laboratory tests, and spirometry are presented in Table 2. In univariate analysis patients with COPD were generally men who smoked more, used more pulmonary medication, had more respiratory symptoms of cough, ABHR, and dyspnoea according to Borg score, and tended to have a lower body mass index. In addition, they had worse lung function test results except for FVC. Other variables studied did not significantly vary between patients with and without COPD based on spirometry.

COPD was not associated with a higher NYHA class (p = 0.130). Also, there were no significant correlations between COPD and LVEF (p = 0.401) or NT-pro-BNP (p = 0.251).

## Discussion

We observed a high prevalence (32.1%) of COPD in a well defined subgroup of stable CHF patients without pulmonary congestion who were recruited from two outpatient cardiology departments of a large general hospital. Using a three month follow-up spirometry to confirm initial diagnosis of de novo COPD did not change COPD prevalence significantly. The majority of patients remained obstructive at follow-up after three month of treatment with tiotropium.

Contrary to our results, initial airway obstruction was found in 19% of patients hospitalised for congestive systolic HF in the study of Brenner et al. [14], but had resolved in 47% of these patients six months after discharge. This indicates that airway obstruction is a dynamic phenomenon in patients with HF, which often resolves after re-compensation. Therefore, a careful timing and interpretation of PFTs is required to avoid misdiagnosis and inappropriate treatment [2]. Ideally, lung function measurements should be obtained under stable conditions when clinically euvolemic to establish a valid diagnosis of COPD. Indeed, the vast majority of patients, except for one (4%), with stable CHF without pulmonary congestion were reproducibly obstructed at follow-up in the current study. There was no indication of asthma in the patient who was not reproducibly obstructed at follow-up.

Our results support previous findings that COPD frequently coexists with HF [15]-[17],[20]-[22], but are in contrast to the study of Brenner et al. [14] who found only 9% of patients with systolic HF to have concomitant COPD, probably explained by the high number of never smokers (45.6%) included in their study. Several factors might explain the high coexistence of these two diseases, including sharing of environmental (mainly smoking) or genetic risk factors, advanced age, systemic inflammation, and a relationship between a reduction in pulmonary and heart function [2],[5]-[7],[29]-[33]. Also, COPD patients are at an increased risk of co-morbidities such as type 2 diabetes, which in turn are an important risk for cardiovascular disease [30]. Furthermore, factors that increase stress on the cardiovascular system or precipitate arrhythmic events can also explain the association between COPD and cardiovascular disease, including hypoxemia, hyperinflation, hyperventilation, neurohumoral disturbances, increased work of breathing and oxygen consumption, pulmonary hypertension, and the use of pulmonary medication [29],[30],[32],[34]. Finally, other factors, such as oxidative stress, endothelial dysfunction, arterial stiffness, and connective tissue degradation have also been suggested to play a role [32],[34].

Since spirometry is still underutilized even in a tertiary-care facility [19], it seems reasonable to consider routine spirometry testing in patients with CHF to diagnose or rule out COPD, a co-morbidity with important therapeutic and prognostic implications [2],[10]-[16],[22] which is still greatly under- and overdiagnosed as found in this study (19% and 32%, respectively). Indeed, diagnostic difficulties have been stressed before, including the overlap in signs, symptoms, and risk factors, the underuse of spirometry despite the fact that objective evidence of airway obstruction is mandatory for diagnosing COPD, and difficulties with interpreting spirometry results, especially in patients with decompensated HF [2],[4]-[7],[14]. This raises concerns regarding possible inappropriate treatment of COPD in an already vulnerable group of patients and as a result possible adverse impact on health and outcome.

Unfortunately, the current study was not large enough to find predictors of newly diagnosed COPD to make specific recommendations regarding which subgroup of patients should be tested. Also, it should be noted that a large proportion (69,2%) of newly diagnosed COPD patients had only mild airway obstruction that may represent a physiological decline of lung function with age instead of a disease [35],[36]. It is unknown whether an additional diagnosis and treatment of COPD in these patients would improve health outcomes and change their prognosis. This warrants further research to establish the effectiveness of screening of patients with CHF for COPD in terms of symptomatic relief and improvement of the outcome as well as cost-effectiveness of such a policy. Until then, spirometry could be used in CHF patients with pulmonary symptoms despite an adequate treatment for their HF, especially in the presence of risk factors for COPD, such as a smoking history of ± 10 PY and occupational exposures. Importantly, spirometry should be used when clinically euvolemic to avoid both misdiagnosis and inappropriate treatment of COPD [2].

The current study has some limitations that deserve further discussion. It is important to realise that the results may not be applicable to all patients with CHF, since we did not include patients with preserved systolic function. The diagnosis of HF with preserved systolic function is challenging and particularly difficult to establish in patients with COPD [5]. Thus, to avoid possible overestimation of COPD prevalence in our population, we only included patients with LVSD. Also, patients with more severe HF could have been under-represented in this study because of inability to participate. COPD prevalence may therefore have been somewhat underestimated. Likewise, there may be a recruitment bias in the cohort, given the refusal of 311 patients to provide informed consent. Finally, another limitation is the relatively small number of patients, particularly with COPD. However, included patient numbers are comparable to other recently published studies [16],[20]-[22].

Despite these limitations, our findings have potential clinical implications. Our results indicate that confirmatory spirometry does not change a newly established diagnosis of COPD in the vast majority of patients with CHF, provided that PFTs are obtained during stable and non-congested conditions. The results also highlight the need for extensive use of spirometry to decrease the burden of undiagnosed or overdiagnosed COPD in patients with CHF. Evidently, the frequent underdiagnosis and overdiagnosis of COPD is not only a concern in the general population, but also in patients who are regularly monitored in outpatient cardiology clinics. Physicians should bear in mind that both diseases often coexist with important diagnostic and therapeutic difficulties and prognostic implications. Thus, both conditions must be simultaneously assessed and collaboration between cardiologists and pulmonologists is essential.

## Conclusions

In conclusion, COPD is a frequent co-morbidity in patients with stable CHF without pulmonary congestion, but is often unrecognized or overdiagnosed. To avoid this and thus ensure adequate treatment of COPD in CHF, PFTs should be routinely obtained in a stable and non-congested condition. Under these conditions a confirmatory spirometry is unnecessary, as it does not change a newly established diagnosis of COPD in the vast majority of patients with CHF.

## Authors’ original submitted files for images

Below are the links to the authors’ original submitted files for images. Authors’ original file for figure 1 Authors’ original file for figure 2

## Abbreviations

ABHR: Aspecific bronchial hyperreactivity
ACE-I: Angiotensin-converting enzyme inhibitor
ARB: Angiotensin receptor blocker
BMI: Body mass index
CABG: Coronary artery bypass grafting
CHF: Chronic HF
COPD: Chronic obstructive pulmonary disease
CRT/ICD: Cardiac resynchronisation therapy
CS: Current smokers
FS: Former smokers
FEV_1_/FVC: Ratio of forced expiratory volume in one second to forced vital capacity
GOLD: Global initiative for chronic obstructive lung disease
HF: Heart failure
ICD: Implantable cardioverter defibrillator
ICS/OCS: Inhalation/oral corticosteroids
LVEF: Left ventricular ejection fraction
LVSD: Left ventricular systolic dysfunction
MLHFQ: Minnesota living with heart failure questionnaire
MRC: Modified Medical Research Council dyspnoea scale
NS: Non-smokers
NT-pro-BNP: N-terminal pro-B natriuretic peptide
NYHA: New York Heart Association
OLD: Obstructive lung disease
PCI: Percutaneous coronary intervention
PFT: Pulmonary function tests
PY: Pack-years
SD: Standard deviation
SPSS: Statistical package for social science

## References

[1] Dickstein K, Cohen-Solal A, Filippatos G, McMurray JJ, Ponikowski P, Poole-Wilson PA, Stromberg A, van Veldhuisen DJ, Atar D, Hoes AW, Keren A, Mebazaa A, Nieminen M, Priori SG, Swedberg K, Vahanian A, Camm J, De Caterina R, Dean V, Dickstein K, Filippatos G, Funck-Brentano C, Hellemans I, Kristensen SD, McGregor K, Sechtem U, Silber S, Tendera M, Widimsky P, Zamorano JL (2008). ESC Guidelines for the diagnosis and treatment of acute and chronic heart failure 2008: the Task Force for the Diagnosis and Treatment of Acute and Chronic Heart Failure 2008 of the European Society of Cardiology. Developed in collaboration with the Heart Failure Association of the ESC (HFA) and endorsed by the European Society of Intensive Care Medicine (ESICM). Eur Heart J.

[2] Hawkins NM, Petrie MC, Jhund PS, Chalmers GW, Dunn FG, McMurray JJ (2009). Heart failure and chronic obstructive pulmonary disease: diagnostic pitfalls and epidemiology. Eur J Heart Fail.

[3] Hannink JD, van Helvoort HA, Dekhuijzen PN, Heijdra YF (2010). Heart failure and COPD: partners in crime?. Respirology.

[4] Rutten FH, Cramer MJ, Lammers JW, Grobbee DE, Hoes AW (2006). Heart failure and chronic obstructive pulmonary disease: an ignored combination?. Eur J Heart Fail.

[5] Le Jemtel TH, Padeletti M, Jelic S (2007). Diagnostic and therapeutic challenges in patients with coexistent chronic obstructive pulmonary disease and chronic heart failure. J Am Coll Cardiol.

[6] Lusuardi M, Garuti G, Massobrio M, Spagnolatti L, Bendinelli S (2008). Heart and lungs in COPD. Close friends in real life-separate in daily medical practice?. Monaldi Arch Chest Dis.

[7] Padeletti M, Jelic S, LeJemtel TH (2008). Coexistent chronic obstructive pulmonary disease and heart failure in the elderly. Int J Cardiol.

[8] Dahlstrom U (2005). Frequent non-cardiac comorbidities in patients with chronic heart failure. Eur J Heart Fail.

[9] Hawkins NM, Jhund PS, Simpson CR, Petrie MC, Macdonald MR, Dunn FG, Macintyre K, McMurray JJ (2010). Primary care burden and treatment of patients with heart failure and chronic obstructive pulmonary disease in Scotland. Eur J Heart Fail.

[10] Rusinaru D, Saaidi I, Godard S, Mahjoub H, Battle C, Tribouilloy C (2008). Impact of chronic obstructive pulmonary disease on long-term outcome of patients hospitalized for heart failure. Am J Cardiol.

[11] Macchia A, Monte S, Romero M, D'Ettorre A, Tognoni G (2007). The prognostic influence of chronic obstructive pulmonary disease in patients hospitalised for chronic heart failure. Eur J Heart Fail.

[12] Staszewsky L, Wong M, Masson S, Barlera S, Carretta E, Maggioni AP, Anand IS, Cohn JN, Tognoni G, Latini R (2007). Clinical, neurohormonal, and inflammatory markers and overall prognostic role of chronic obstructive pulmonary disease in patients with heart failure: data from the Val-HeFT heart failure trial. J Card Fail.

[13] De Blois J, Simard S, Atar D, Agewall S (2010). COPD predicts mortality in HF: the Norwegian Heart Failure Registry. J Card Fail.

[14] Brenner S, Guder G, Berliner D, Deubner N, Frohlich K, Ertl G, Jany B, Angermann CE, Stork S (2013). Airway obstruction in systolic heart failure - COPD or congestion?. Int J Cardiol.

[15] Arnaudis B, Lairez O, Escamilla R, Fouilloux A, Fournier P, Monteil B, Bouisset F, Arnal JF, Elbaz M, Carrie D, Roncalli J, Pathak A, Galinier M (2012). Impact of chronic obstructive pulmonary disease severity on symptoms and prognosis in patients with systolic heart failure. Clin Res Cardiol.

[16] Mascarenhas J, Lourenco P, Lopes R, Azevedo A, Bettencourt P (2008). Chronic obstructive pulmonary disease in heart failure. Prevalence, therapeutic and prognostic implications. Am Heart J.

[17] Iversen KK, Kjaergaard J, Akkan D, Kober L, Torp-Pedersen C, Hassager C, Vestbo J, Kjoller E (2008). Chronic obstructive pulmonary disease in patients admitted with heart failure. J Intern Med.

[18] Rabe KF, Hurd S, Anzueto A, Barnes PJ, Buist SA, Calverley P, Fukuchi Y, Jenkins C, Rodriguez-Roisin R, van Weel C, Zielinski J (2007). Global strategy for the diagnosis, management, and prevention of chronic obstructive pulmonary disease: GOLD executive summary. Am J Respir Crit Care Med.

[19] Damarla M, Celli BR, Mullerova HX, Pinto-Plata VM (2006). Discrepancy in the use of confirmatory tests in patients hospitalized with the diagnosis of chronic obstructive pulmonary disease or congestive heart failure. Respir Care.

[20] Steinacher R, Parissis JT, Strohmer B, Eichinger J, Rottlaender D, Hoppe UC, Altenberger J (2012). Comparison between ATS/ERS age- and gender-adjusted criteria and GOLD criteria for the detection of irreversible airway obstruction in chronic heart failure. Clin Res Cardiol.

[21] Boschetto P, Fucili A, Stendardo M, Malagu M, Parrinello G, Casimirri E, Potena A, Ballerin L, Fabbri LM, Ferrari R, Ceconi C (2013). Occurrence and impact of chronic obstructive pulmonary disease in elderly patients with stable heart failure. Respirology.

[22] Macchia A, Rodriguez Moncalvo JJ, Kleinert M, Comignani PD, Gimeno G, Arakaki D, Laffaye N, Fuselli JJ, Massolin HP, Gambarte J, Romero M, Tognoni G (2012). Unrecognised ventricular dysfunction in COPD. Eur Respir J.

[23] Minasian AG, van den Elshout FJ, Dekhuijzen PN, Vos PJ, Willems FF, van den Bergh PJ, Heijdra YF (2013). COPD in chronic heart failure: less common than previously thought?. Heart Lung.

[24] O'Hanlon R, O'Shea P, Ledwidge M, O'Loughlin C, Lange S, Conlon C, Phelan D, Cunningham S, McDonald K (2007). The biologic variability of B-type natriuretic peptide and N-terminal pro-B-type natriuretic peptide in stable heart failure patients. J Card Fail.

[25] Rector TS, Kubo SH, Cohn JN (1987). Patients' self-assessment of their congestive heart failure. Part 2: content, reliability and validity of a new measure, the Minnesota Living with Heart Failure questionnaire. Heart Failure.

[26] Bestall JC, Paul EA, Garrod R, Garnham R, Jones PW, Wedzicha JA (1999). Usefulness of the Medical Research Council (MRC) dyspnoea scale as a measure of disability in patients with chronic obstructive pulmonary disease. Thorax.

[27] Borg GA (1982). Psychophysical bases of perceived exertion. Med Sci Sports Exerc.

[28] Quanjer PH, Tammeling GJ, Cotes JE, Pedersen OF, Peslin R, Yernault JC (1993). Lung volumes and forced ventilatory flows. Report working party standardization of lung function tests, European community for steel and coal. Official statement of the European Respiratory Society. Eur Respir J.

[29] Sin DD, Man SF (2005). Chronic obstructive pulmonary disease: a novel risk factor for cardiovascular disease. Can J Physiol Pharmacol.

[30] Finkelstein J, Cha E, Scharf SM (2009). Chronic obstructive pulmonary disease as an independent risk factor for cardiovascular morbidity. Int J Chron Obstruct Pulmon Dis.

[31] Barr RG, Bluemke DA, Ahmed FS, Carr JJ, Enright PL, Hoffman EA, Jiang R, Kawut SM, Kronmal RA, Lima JA, Shahar E, Smith LJ, Watson KE (2010). Percent emphysema, airflow obstruction, and impaired left ventricular filling. N Engl J Med.

[32] Malerba M, Romanelli G (2009). Early cardiovascular involvement in chronic obstructive pulmonary disease. Monaldi Arch Chest Dis.

[33] Malerba M, Ragnoli B, Salameh M, Sennino G, Sorlini ML, Radaeli A, Clini E (2011). Sub-clinical left ventricular diastolic dysfunction in early stage of chronic obstructive pulmonary disease. J Biol Regul Homeost Agents.

[34] Macnee W, Maclay J, McAllister D (2008). Cardiovascular injury and repair in chronic obstructive pulmonary disease. Proc Am Thorac Soc.

[35] Hardie JA, Buist AS, Vollmer WM, Ellingsen I, Bakke PS, Morkve O (2002). Risk of over-diagnosis of COPD in asymptomatic elderly never-smokers. Eur Respir J.

[36] Enright P, Brusasco V (2010). Counterpoint: should we abandon FEV/FVC < 0.70 to detect airway obstruction? Yes. Chest.

