# Supplementary figures and images for: Serial pulmonary function tests to diagnose COPD in chronic heart failure
Source: Transl Respir Med. 2014 Sep 25;2:12. doi: 10.1186/s40247-014-0012-5 (PMC4177105; doi:10.1186/s40247-014-0012-5)

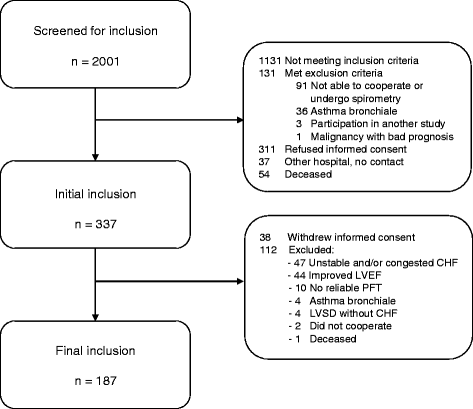

Supplement: Supplementary file 1 — Authors’ original file for figure 1 [file 40247_2014_12_MOESM1_ESM.gif]

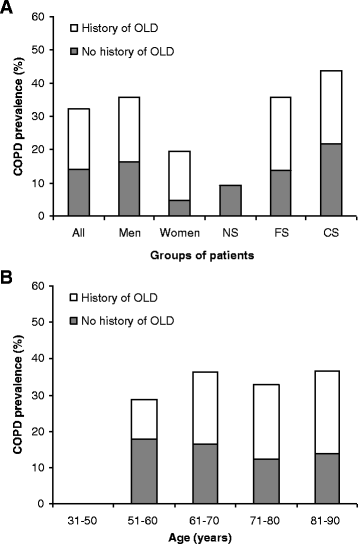

Supplement: Supplementary file 2 — Authors’ original file for figure 2 [file 40247_2014_12_MOESM2_ESM.gif]
